# Genomic epidemiology of erythromycin-resistant *Bordetella pertussis* in China

**DOI:** 10.1080/22221751.2019.1587315

**Published:** 2019-03-22

**Authors:** Zheng Xu, Zengguo Wang, Yang Luan, Yarong Li, Xiaoguai Liu, Xiaokang Peng, Sophie Octavia, Michael Payne, Ruiting Lan

**Affiliations:** a School of Biotechnology and Biomolecular Sciences, University of New South Wales, Sydney, Australia; b Xi’an Center for Disease Prevention and Control, Xi’an, People’s Republic of China; c Department of Infectious Diseases, Xi’an Children’s Hospital, Xi’an, People’s Republic of China

**Keywords:** *Bordetella pertussis*, erythromycin-resistant, genomic epidemiology, *fhaB*, *ptxP1*

## Abstract

Macrolides such as erythromycin are the empirical treatment of *Bordetella pertussis* infections. China has experienced an increase in erythromycin-resistant *B. pertussis* isolates since they were first reported in 2013. Here, we undertook a genomic study on Chinese *B. pertussis* isolates from 2012 to 2015 to elucidate the origins and phylogenetic relationships of erythromycin-resistant *B. pertussis* isolates in China. A total of 167 Chinese *B. pertussis* isolates were used for antibiotic sensitivity testing and multiple locus variable-number tandem repeat (VNTR) analysis (MLVA). All except four isolates were erythromycin-resistant and of the four erythromycin-sensitive isolates, three were *non-ptxP1*. MLVA types (MT), MT55, MT104 and MT195 were the predominant types. Fifty of those isolates were used for whole genome sequencing and phylogenetic analysis. Genome sequencing and phylogenetic analysis revealed three independent erythromycin-resistant lineages and all resistant isolates carried a mutation in the 23S rRNA gene. A novel *fhaB3* allele was found uniquely in Chinese *ptxP1* isolates and these Chinese *ptxP1-ptxA1-fhaB3* had a 5-fold higher mutation rate than the global *ptxP1-ptxA1 B. pertussis* population. Our results suggest that the evolution of Chinese *B. pertussis* is likely to be driven by selection pressure from both vaccination and antibiotics. The emergence of the new non-vaccine *fhaB3* allele in Chinese *B. pertussis* population may be a result of selection from vaccination, whereas the expansion of *ptxP1-fhaB3* lineages was most likely to be the result of selection pressure from antibiotics. Further monitoring of *B. pertussis* in China is required to better understand the evolution of the pathogen.

## Introduction

Pertussis (whooping cough) is caused by the Gram-negative bacterium *Bordetella pertussis*. Macrolides, especially erythromycin, are the empiric treatment for pertussis. Although the first erythromycin-resistant case was found in the United States in 1994 [1], only a few resistant pertussis cases have been reported globally [2,3]. However, China has seen a rapid increase in erythromycin-resistant *B. pertussis* since the first case reported in 2013 [4–6]. There are two known mechanisms for resistance to erythromycin: the acquisition of erythromycin-resistant methylase (*erm*) genes resulting in high-level resistance [7], and mutations in the 23S rRNA gene leading to structural changes that prevent the binding of erythromycin [2]. Previous studies of erythromycin-resistant *B. pertussis* isolates in China showed that all resistant isolates contained 23S rRNA gene mutations [5,8]. Moreover, these erythromycin-resistant isolates are also resistant to other macrolides [4,6].

Whole cell vaccine (WCV) against pertussis was introduced in China in early 1960s. Two component [pertussis toxin (Ptx) and filamentous haemagglutinin (Fha)] acellular pertussis vaccine (ACV) started to replace WCV in 2007 and completely replaced WCV in 2012 [6]. The national immunization programme for pertussis in China recommends three primary doses of DTaP (diphtheria, tetanus and acellular pertussis) at 3, 4 and 5 months and a booster at 18–24 months of age [9]. Despite over 99% vaccine coverage in children in China, *B. pertussis* infection is still very common among children, adolescent and adults [10].

Although pertussis is a vaccine-preventable disease, the disease still imposes a heavy burden on global public health. Different types of ACV with 1–5 components were used by different countries [9,11,12]. Ptx is the major virulence factor in *B. pertussis* and one of the main components in ACV. It has been suggested that ACV-induced selection pressure results in polymorphisms on the genes encoding ACV components [13]. To date, 18 pertussis toxin promoter (*ptxP*) alleles have been identified but only two, *ptxP1* and *ptxP3*, were commonly observed. Since early 2000, isolates carrying the *ptxP3* allele became the predominant *ptxP* type in many countries, including the Netherlands, Finland and Australia [13–15]. By contrast, the Chinese pertussis isolates predominantly carried the *ptxP1* allele [16,17].

In this study, we aimed to elucidate the genomic evolution of erythromycin-resistant Chinese *B. pertussis* isolates from 2012 to 2015 and evaluate their molecular epidemiological characteristics.

## Material and methods

### Bacterial isolates and susceptibility to antimicrobials

A total of 167 isolates were collected according to Chinese clinical case diagnostic criteria for pertussis from 5 provinces in the Midwest of China between 2012 and 2015 (Supplementary Table1). Isolates were cultured on Bordet-Gengou agar (Oxoid) supplemented with 10% horse blood at 37°C for 3–5 days and further tested by E-test for resistance to erythromycin. *Staphylococcus aureus* ATCC25923 and *B. pertussis* ATCC9797 were used as controls.

### Multiple locus variable-number tandem repeat (VNTR) analysis (MLVA)

MLVA was performed following the procedures described by Schouls et al. [18]. Five loci (VNTR1, VNTR3, VNTR4, VNTR5 and VNTR6) were amplified by PCR and the MLVA type (MT) was assigned using the *B. pertussis* MLVA database at https://www.mlva.net/. The minimum spanning tree was generated using GrapeTree [19].

### Isolates selection and whole genome sequencing

**Figure 1.**
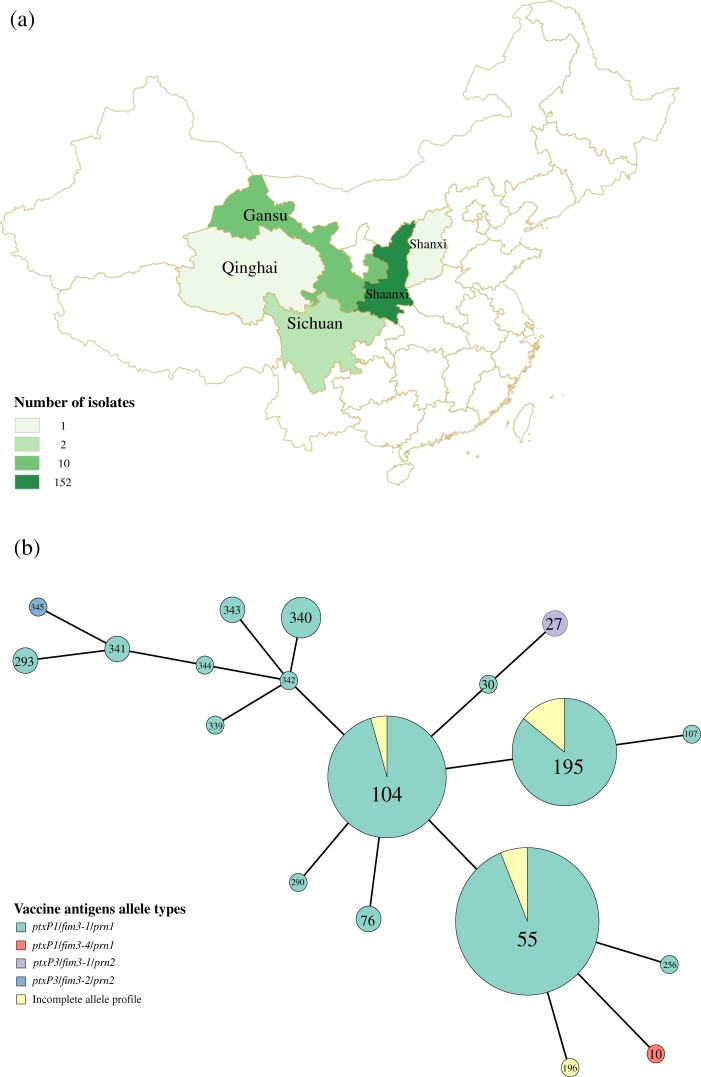
(a) The geographical distribution of the 167 *B. pertussis* isolates. Regions were coloured by different shades proportional to the number of isolates from a given region as shown in the legend. (b) Minimum spanning tree of MLVA types of the 167 *B. pertussis* isolates collected between 2013-2015. Each circle represents an MLVA type (MT) with the number in the circle. The circle size is proportional to the number of isolates belonging to the particular MT. The fill colour of the circles indicates the different allelic profiles of vaccine antigen genes.

Real-time PCR targeting IS*481* and *ptxP* were performed for all 167 suspected *B. pertussis* isolates. All isolates were found to be positive for both gene targets [20]. Fifty of the 167 isolates were sequenced by Illumina paired end sequencing which included three *ptxP3* isolates and 47 *ptxP1* isolates representing different MTs (MT10, 27, 55, 76, 104, 195, 196, 256, 290, 293) from different years (2012, 2013, 2014 and 2015) and collected from 20 different cities in 5 provinces (Figure 1(a)). Genomic DNA was extracted by the Wizard Genomic DNA Purification Kit (Promega). The isolates were sequenced using Illumina HiSeq and reads were assembled *de novo* using SPAdes version 3.7.0 [21] (Supplementary Table2). Genomic DNA libraries were prepared using the KAPA HyperPlus Kit (Roche) and sequenced using HiSeq X Ten Reagent Kit v2.5 (Illumina). Raw reads were submitted to GenBank under the BioProject PRJNA489102.

### Analysis of vaccine antigen genes, 23S rRNA genes, and erythromycin ribosomal methylase (erm) genes

PCR was used for typing the vaccine antigen genes, *ptxP*, *fim3* and *prn* [22–24]. For isolates with sequenced genomes, *in silico* typing of the vaccine antigen genes, *ptxP*, *ptxA*, *prn*, *fim2*, *fim3*, and screening for 23S rRNA mutation and the presence of the *erm* genes [25] were performed using BLAST+ tools (version 2.6.0). The reported A2047G position is based on an old Tohama I 23S rRNA sequence [2] and is equivalent to A2037G in the current Tohama I genome (Accession No. NC_002929.2). To be consistent with published work, we use the A2047G convention as position reference throughout the text.

### Single nucleotide polymorphism (SNP) detection and phylogenetic analysis

SNP detection used a combination of mapping by Burrow-Wheeler Alignment (BWA) tool version 0.7.12 [26] and *de novo* assembly using SPAdes (version 3.7.0) [21]. SAMtools version 0.1.19 [27] was used for SNP calling. IGV version 2.3.94 was used to manually inspect read alignments to confirm SNP calls. Alignment of *de novo* assemblies was performed using progressiveMauve (version snapshot_2015_02-25). Phylogenetic trees were constructed using MEGA version 5.2.1, the maximum parsimony tree was applied based on the Tree-bisection-Reconnection (TBR) method and the bootstrap analysis was based on 1000 replicates (SNPs files shown in Supplementary Table3). Short insertions/deletions (indels) (less than 100 bp) were identified by SAMtools. Determination of IS*481* and IS*1002* insertion was performed using a custom script as previously described in Safarchi et al. [28] and Octavia et al. [29]. Estimation of the nucleotide substitution rate for each site was performed using Bayesian analysis by the BEAST package version 1.8.4 [30]. The uncorrelated relaxed clock with constant population size model was found to be the most appropriate for Chinese *B. pertussis* lineages. The IEDB database (https://www.iedb.org/) [31] was used for Fha epitope searching and the program PROVEAN (provean.jcvi.org/index.php) [32] was used for predicting the effect of amino acid change.

## Ethics

This study was approved by the Institutional Review Board of the Xi’an Center for Disease Control and Prevention, Xi’an, China.

## Results

### Genotypic analysis and antibiotic testing of *B. pertussis* isolates

A total of 167 isolates were collected from 2012 to 2015, from Midwest China (Figure 1(a), Supplementary Table 1). Of these, 163 isolates had an erythromycin minimum inhibitory concentration (MIC) >256 IU/L suggesting erythromycin-resistance phenotype while the remaining four isolates were sensitive to erythromycin. Of these four erythromycin-sensitive isolates, one was a *ptxP1* isolate and three were *ptxP3* isolates.

Genotypic typing of vaccine antigen genes by PCR sequencing [24] showed that 156 were *ptxP1*/*fim3-1*/*prn1*, *ptxP1*/*fim3-4*/*prn1,* while 3 were *ptxP3*/*fim3-1*/*prn2*, *ptxP3*/*fim3-2*/*prn2*. The remaining isolates contained incomplete allelic profiles (Figure 1(b)). MLVA was performed on these isolates. Twelve MTs were observed of which seven were new (MT339-MT345). MT55, MT104 and MT195 were the predominant MTs, all of which except the incomplete allele profiles had *ptxP1* allele. Of the three *ptxP3* isolates, two belonged to MT27, a common *ptxP3* MT, and one was a new type.

### Whole genome sequencing of selected isolates

Fifty isolates including 47 *ptxP1* isolates and three *ptxP3* isolates were sequenced. Using Tohama I as the reference genome, the number of SNPs varied from 192 to 253 per isolate. Among the 47 *ptxP1* Chinese isolates, 517 SNPs were detected, of which 33.46% (173/517) were non-coding (nc) SNPs (ncSNPs) located in intergenic (IG) regions, 21.86% (113/517) synonymous (s) SNPs (sSNPs) and 37.14% (192/517) non-synonymous (ns) SNPs (nsSNPs). Note that the three *ptxP3* isolates were found to be distributed amongst global *ptxP3* strains and were not analysed further (Supplementary Figure1).

**Figure 2.**
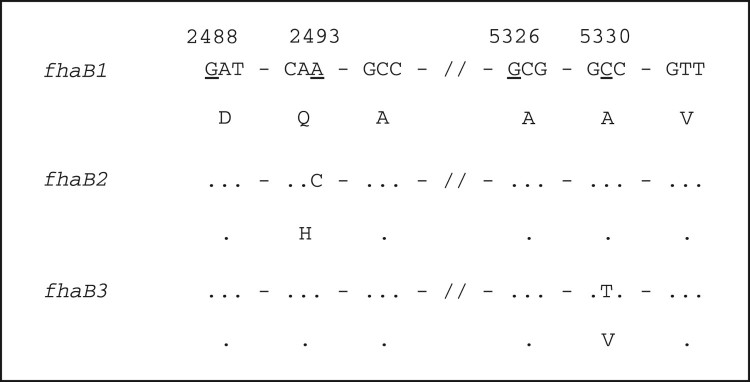
Sequence difference of different *fhaB* alleles. Numbers at the top refer to the position of the underline nucleotide, which is relative to the start of *fhaB* gene. Dots indicate identity. Nucleotide sequence is shown in codons with amino acid in single letter shown below.

*In silico* analysis of the vaccine antigen genes was consistent with PCR sequencing results. Additionally, a mutation in position 5330 of the *fhaB* gene that resulted in the amino acid changing from alanine to valine was found in 46 isolates. Following the convention for *fhaB* allele naming by van Loo et al. [33] which defined *fhaB1* and *fhaB2* alleles, we designated the novel allele with C5330 T mutation as *fhaB*3 (GenBank Accession No. MH824675) (Figure 2). The mutated region was in one of the Fha epitopes according to the database IEDB (https://www.iedb.org/), which is related to the MHC class I. PROVEAN (http://provean.jcvi.org/index.php) predicting showed that the substitution does not affect protein structure. However, it may still affect immune recognition.

All genomes were searched for the presence of *erm* genes, but none were found [25]. Instead, the 46 erythromycin-resistant isolates contained a A2047G mutation in the domain V region of the 23S rRNA genes. *B. pertussis* has 3 copies of the *rrn* operon. No heterogenous bases were detected at 2047 site of the 23S rRNA gene in any of the 46 erythromycin-resistant isolates, suggesting that all three *rrn* operons carried the A2047G mutation.

### Phylogenetic analysis of erythromycin-resistant Chinese *B. pertussis* isolates

**Figure 3.**
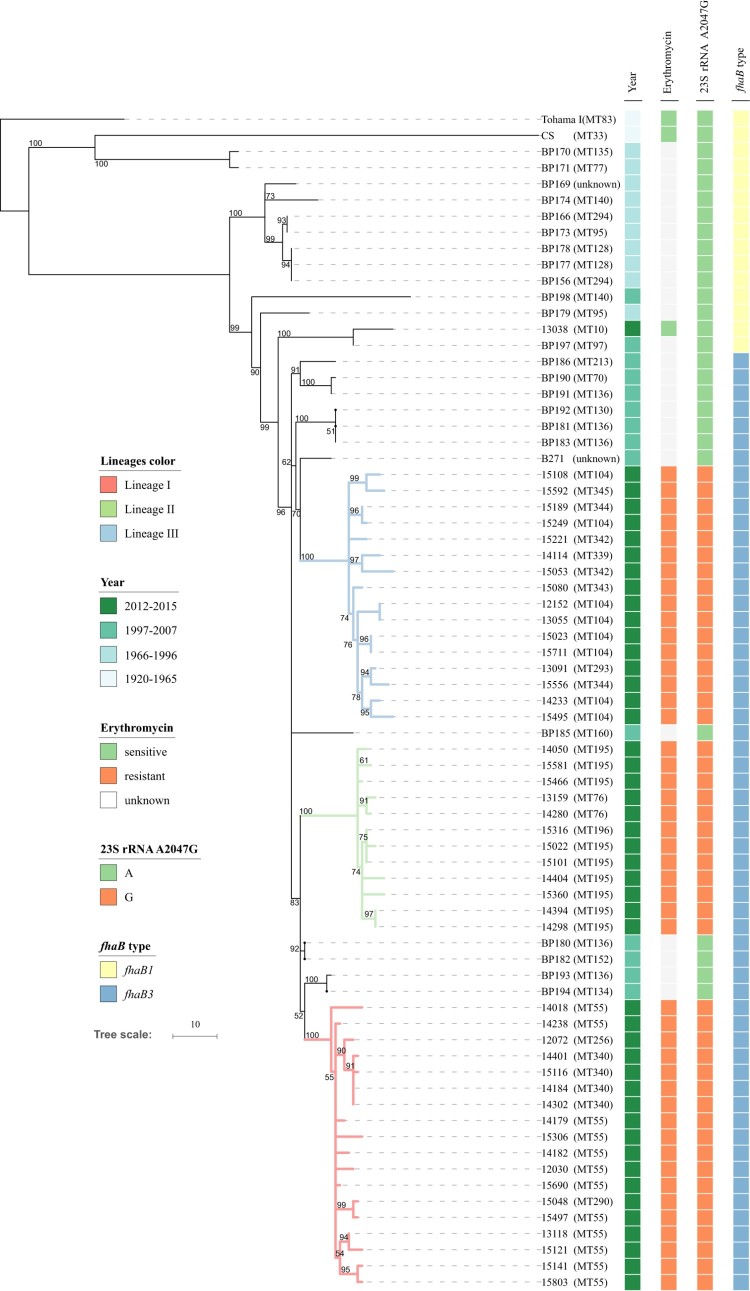
Phylogenomic relationship of 71 Chinese *B. pertussis* isolates. A maximum parsimony tree was generated based on 928 SNPs to illustrate the genetic relationship of erythromycin-resistant isolates. Tohama I was used as an outgroup. The three erythromycin-resistant lineages I, II, and III were marked by red, green and blue respectively. Bootstrap values of ≥50% were marked on each branch. The unit bar represents 10 SNPs. Isolate details (year, phenotype of and genotype of erythromycin-sensitivity and *fhaB* allele type) are shown as colour codes per the legends. MLVA types are shown in the brackets following the isolate's name.

A total of 71 Chinese *B. pertussis* isolates were used for phylogenetic analysis, including 47 *ptxP1* isolates from this study and 24 *ptxP1* isolates from a previous Chinese study [34] (Figure 3). Tohama I was used as the outgroup. The 46 erythromycin-resistant isolates were grouped into three lineages: Lineage I (18 isolates), Lineage II (12 isolates) and Lineage III (16 isolates), which were supported by five [one sSNP, one nsSNP and three ncSNPs], 12 (three sSNPs, five nsSNPs and four ncSNPs) and nine SNPs (three sSNPs, five nsSNPs and one ncSNP) (Supplementary Table4), respectively. Each of the three lineages were supported by a bootstrap value of 100% with 1000 replicates.

Lineage I was grouped together with 2 erythromycin-sensitive isolates BP193 and BP194, supported by one nsSNP. Lineages I and II were closely related, supported by two SNPs (one ncSNP and one sSNP), and grouped together with two erythromycin-sensitive isolates, BP180 and BP182. They were clearly separated from Lineage III, supported by a bootstrap value of 83%. Lineage III shared one sSNP with erythromycin-sensitive isolates BP181, BP183 and BP192 as well as an erythromycin-sensitive isolate from Taiwan (B271), supported by a bootstrap value of 62%. Bootstrap value for the erythromycin-resistant branches with interspersed erythromycin-sensitive isolates was 96%. These high bootstrap values support the inference that these erythromycin-resistance lineages were derived from erythromycin-sensitive isolates and erythromycin-resistance arose independently three times.

### Relationships of erythromycin-resistant Chinese isolates with global *ptxP1* isolates

**Figure 4.**
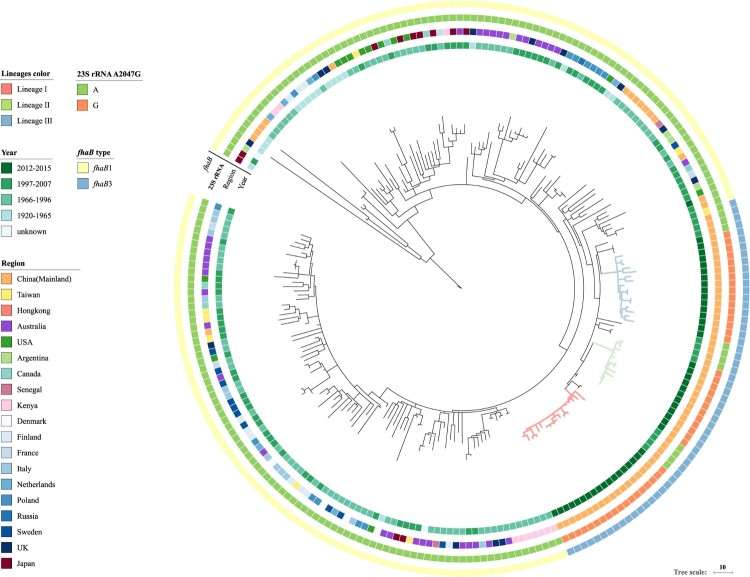
Genomic relationship of global *ptxP1* strains. The tree was made using 235 *B. pertussis* genomes including the 47 Chinese *ptxP1* strains sequenced in this study and was based on 2744 SNPs by the maximum parsimony method. Three erythromycin-resistant lineages I, II, and III were marked by red, green and blue respectively. Tohama I was used as the outgroup. The unit bar represents 10 SNPs. Isolate details (year, region, genotype of erythromycin-sensitvity and *fhaB* allele type) are shown as colour codes as per the legends.

We expanded our genome analysis to include 162 global *ptxP1* isolates from Bart et al*.* study [15] (Figure 4). The Chinese erythromycin-resistant isolates and 12 other Chinese erythromycin-sensitive isolates from 1997 to 2007 were grouped together exclusively without isolates from other regions, showing that the erythromycin-resistant lineages arose out of the erythromycin-sensitive isolates in China. There were three SNPs supporting this group (located in genes BP1879, BP3362 and BP3466). We named this group as the *fhaB3* lineage as all isolates within the group had the *fhaB3* allele (Figure 4). No *ptxP1* isolates, other than the 46 Chinese isolates from this study, had the A2047G mutation in the 23S rRNA gene suggesting that they were all erythromycin-sensitive. BEAST analysis showed the three clusters arose at different times: Lineage III arose around 2003 while Lineage I and II arose around 2008 and 2007, respectively.

### Insertion sequence (IS) elements

*B. pertussis* genome has more than 200 copies of insertion sequence (IS) elements [35]. In the *B. pertussis* reference genome, Tohama I, it contains 238 and six copies of IS*481*, IS*1002* respectively [36]. In the 50 genomes from this study, the average copy number of IS*481* and IS*1002* was 197 and five, respectively. There was no unique IS insertion common to all erythromycin-resistant isolates. IS elements inserted in the *prn* gene have been shown to result in Prn negative isolates [12]. One isolate (15221) was found to have an IS*481* inserted at position 1598 in its *prn* gene, and thus was likely to be a Prn negative isolate.

### Mutation rate

A previous study suggested different lineages may have different mutation rates. We calculated the mutation rate of the *fhaB3* lineage and the mutation rate of *ptxP1-ptxA1* strains from the global isolates excluding the *fhaB3* isolates for comparison. Note that nearly all isolates were *ptxA1* after the introduction of WCV in the 1950s and the genetic diversity of the *B. pertussis* population was reduced with the replacement of previously prevalent *ptxA2* strains by *ptxA1* strains [14]. The mutation rate of the *fhaB3* lineage was 7.22 × 10^–3^ (95%CI 4.78 × 10^–3^, 9.87 × 10^–3^), which was 1.21 × 10^–6^ substitution/site/year using a core genome size of 3,485,486 bp [36], while the mutation rate of the global non-*fhaB3* isolates was 5.39 × 10^–4^ (95%CI 4.48 × 10^–4^, 6.34 × 10^−4^) which translates to 2.11 × 10^–7^ substitution/site/year. Our estimate is similar to the earlier estimates of the Chinese *B. pertussis* population by Xu et al. [34] of 3.06 × 10^–6^ substitution/site/year, confirming that the Chinese *ptxP1* lineage evolves faster than the global *ptxP1* population. Note that the mutation rate of 2.24 × 10^–7^ substitution/site/year by Bart et al. [15] included *ptxP3* strains and is an overall rate. We examined mutator genes for any mutations [37] that may have contributed to the increased mutation rate. No SNPs were located in any of the mutator genes examined.

## Discussion

This study has shown that *ptxP1 B. pertussis* strains continue to be more prevalent in China as 98.11% (156/159) of the isolates carried *ptxP1* allele, which is different from the global trend where *ptxP3* strains were more prevalent [15,16]. The vast majority of the isolates (97.6% [163/167]) were identified as erythromycin-resistant, showing rapid expansion of erythromycin-resistant strains in China.

Erythromycin-resistant Lineages I, II and III were represented predominantly by MT55, MT195 and MT104, respectively (Figure1 (b)). The three major MTs types were different from a previous study, which showed that the MLVA types of Chinese isolates have changed continuously in different periods. However, it is also possible that different *B. pertussis* populations circulate in different regions of China as most isolates in Xu et al*.* study were from Beijing in the North of China while isolates from this study were from Midwestern regions. Xu et al. also showed that the MLVA types of Chinese isolates changed dynamically in different periods [16].

Non-expression of Prn has been reported in many countries where ACV containing Prn was used and high prevalence of Prn deficient strains have been reported in Australia and some other countries [12,38–40]. Interestingly, in this study, one isolate (15221) was found to have IS*481* inserted in its *prn* gene. The insertion was at a location known to cause Prn inactivation and thus the isolate is most likely not expressing Prn. This Prn inactivation is unlikely to be due to selection pressure from the Chinese ACV as it contains no Prn. However, the emergence of the first Prn-negative isolate, which occurred in an erythromycin*-*resistant *ptxP1* background, raises concerns of such isolates spreading to countries where ACVs containing Prn are used since higher fitness was shown for Prn negative strains in a mouse model [41].

Phylogenetic analysis showed that erythromycin-resistant isolates in this study evolved as three independent lineages from erythromycin-sensitive strains. The three lineages were well supported by multiple SNPs. Each lineage was grouped with erythromycin-sensitive isolates, supported by one to two SNPs. Although it is possible that a SNP may arise independently, there have been no parallel mutations observed in *B. pertussis* except in vaccine antigen genes [38]. The SNPs supporting the grouping of sensitive isolates with Lineage I (five) and Lineage II (12) were located in ncSNPs or sSNPs and thus less likely to be under positive selection pressure. Further, Lineages I and II, together with closely related erythromycin-sensitive isolates were well separated from Lineage III, providing a firm support of independent origin of these lineages.

All Chinese *fhaB3* formed a separate lineage that contained only Chinese isolates, suggesting that this mutation arose once and expanded in China. The *fhaB3* mutation arose in early 1990s from BEAST estimates, before ACV was introduced in China but ACV may have selected for the new allele. As the Chinese vaccine strain CS contains *fhaB1* allele, the emergence and expansion of *fhaB3* allele may be a result of selection pressure from vaccination. Additionally, the expansion of *fhaB3* isolates may also be due to the selection pressure from antibiotics as the majority of the *fhaB3* isolates from this study were resistant to erythromycin. Further studies are required to determine whether this mutation confers any advantage as Fha is one of the two components (Ptx and Fha) of the Chinese ACV. It is important to note that the Chinese vaccine strain CS contained *ptxA2-fhaB1* alleles while the Chinese *ptxP1 B. pertussis* population carried *ptxA1-fhaB3* alleles, causing mismatch in both vaccine antigen alleles.

The Chinese *ptxP1* strains formed a single lineage which also uniquely carried the *fhaB3* allele. The mutation rate of the Chinese *ptxP1-ptxA1-fhaB3* lineage was five times higher than the rest of the global *ptxP1*-*ptxA1* isolates, consistent with an earlier estimate. The genetic basis of increased mutation rate is unknown as there were no SNPs in any of the mutator genes that may increase mutation rate [37]. The increased mutation rate may have played a role in the adaptation of Chinese *B. pertussis* against antibiotic-driven selection pressure and/or WCV selection pressure.

The rise of erythromycin/azithromycin resistance in *B. pertussis* in China could be from direct or indirect selection pressure from the use of macrolides. Sixteen isolates included in this study have previously been shown to be resistant to azithromycin by Li et al. [42], suggesting that the erythromycin-resistant isolates in this study were also resistant to azithromycin. Macrolides are the primary therapeutic choice of both pertussis and *Mycoplasma pneumoniae* associated pneumonia. Macrolide-resistant *M. pneumoniae* in China has been previously reported with prevalence of resistance from 69% to 95% [43,44]. It is likely that the frequent use of erythromycin has contributed to the emergence and spread of both erythromycin-resistant *M. pneumoniae* and *B. pertussis*. There are few reports of erythromycin-resistant *B. pertussis* [3,5,45,46]. Resistance is low or none in European countries [47], Australia [48] and the US [49] where the use of antibiotics is controlled and guided by microbiological diagnosis. It will be interesting to see how the *B. pertussis* population evolves in countries where there is a similar high usage of antibiotics to China. However, many developing countries still use the whole cell vaccine, where the selection pressures may be different from what we have seen in China.

It is intriguing that all erythromycin-resistant isolates had a high MIC of >256 μg/ml with no intermediate level of resistance observed. Lower resistance has been reported previously in two US isolates with one each displaying MIC values of 32 and 64 μg/ml [3,50]. The resistance in the Chinese isolates has been attributed to the A2047G mutation in the 23S rRNA gene while the mechanism of resistance in the US isolates is unknown. We found no other erythromycin resistance genes present in the genomes of the Chinese isolates. The presence of the resistance conferring A2047G mutation in all 3 copies of the 23S rRNA may have conferred the high level of erythromycin resistance. A likely explanation of how all 3 copies of the 23S rRNA gene shared the same mutation is homologous recombination between the rRNA operons. The A2047G mutation may have initially appeared in one copy of the 23S rRNA due to antibiotic selection and then recombined into the other 2 copies [51].

The Chinese *B. pertussis* population predominantly carried the *ptxP1* allele. It has been observed previously that there is little geographical separation of *B. pertussis* population [15,29]. Overall, *ptxP3 B. pertussis* circulates in countries where ACV has been in use while WCV countries have been dominated by *ptxP1* strains [52]. The replacement of WCV in China in 2012 occurred a decade later than developed countries such as the US and Australia [6]. Therefore, there may not have been enough time for *ptxP3* strains to replace *ptxP1* strains in China. However, *ptxP3* strains were predominant before ACV was introduced in some countries such as the US [13]. Additionally, *ptxP3* strains are fitter than *ptxP1* strains regardless of ACV immunization in a mixed infection mouse model [53] and thus are expected to outcompete *ptxP1* strains in its natural hosts. The predominance of *ptxP1* strains in China may be a result of selection from antibiotics as the erythromycin-resistant strains clearly have had an advantage over erythromycin-sensitive *ptxP3* strains in a host population where antibiotics are known to be overused. Therefore, antibiotic-driven selection pressure may play a greater role in the evolution of *B. pertussis* isolates than vaccine in China and help maintain a separate *B. pertussis* population.

In addition to the much later introduction of ACV, China also uses an ACV with different ACV components and a different vaccine schedule. The Chinese national immunization programme recommends three primary doses of DTaP at 3, 4 and 5 months and a booster at 18–24 months [9]. No pertussis booster doses are administered for primary school age children as the booster given to 6-year-old children only includes diphtheria and tetanus toxoids without the acellular pertussis component [9]. Given the differences in vaccine and antibiotic selection pressures imposed on the Chinese *B. pertussis* population, we expect a different evolutionary trajectory of *B. pertussis* in China.

## Conclusion

In conclusion, the evolution of Chinese *B. pertussis* is likely to be driven by selection pressure from both vaccination and antibiotics, which may have maintained the unique *B. pertussis* population in China. The potential spread of the erythromycin-resistant Chinese *ptxP1* strains to other countries is also of global public health concern, especially if they gain a competitive advantage over *ptxP3* strains currently circulating more globally. Further monitoring of *B. pertussis* in China is required to better understand the evolution of the pathogen.

## Supplementary Material

Supplemental Material
