# Supplementary material for: Genomic epidemiology of erythromycin-resistant Bordetella pertussis in China
Source: Emerg Microbes Infect. 2019 Mar 22;8(1):461–70. doi: 10.1080/22221751.2019.1587315 (PMC6455148; doi:10.1080/22221751.2019.1587315)
Supplement: Supplemental Material [file TEMI_A_1587315_SM3634.zip › Supplementary Material/TEMI-2019-0115/Supplementary_Figure1_legend.docx]

**Supplementary Figure 1.** Phylogenetic relationship of the three Chinese *ptxP3* isolates with the global *ptxP3* isolates. The tree constructed using the maximum parsimony method in MEGA. The isolates name labeled in red color are the *ptxP3* isolates from our study.
